# TECHNIQUE OF EXPOSURE OF THE ESOPHAGOGASTRIC JUNCTION OBTAINED BY THE FLEXIBLE LIVER RETRACTOR IN BARIATRIC SURGERY: A RANDOMIZED CONTROLLED TRIAL

**DOI:** 10.1590/0102-672020210002e1631

**Published:** 2022-01-31

**Authors:** Rodrigo Feitosa de Albuquerque Lima BABADOPULOS, Luiz Gonzaga de MOURA-JR, Vagnaldo FECHINE, Marina Becker Sales ROCHA, Natalícia ANTUNES, Thomaz Alexandre COSTA, Bruno Almeida COSTA, Manoel Odorico DE-MORAES

**Affiliations:** 1 Unidade de Cirurgia Bariátrica, Hospital Geral Dr. César Cals, Fortaleza, CE, Brasil; 2 Centro de Pesquisa e Desenvolvimento de Medicamentos, Unidade de Farmacologia Clínica, Universidade Federal do Ceará, Fortaleza, CE, Brasil; 3 Departamento de Farmacologia, Faculdade de Ciências Médicas, Universidade Estadual de Campinas - Unicamp, Campinas, São Paulo - SP, Brasil

**Keywords:** Bariatric surgery, Esophagogastric junction, Obesity, Laparoscopy, Gastric bypass, Cirurgia bariátrica, Junção esofagogástrica, Laparoscopia, Obesidade, Derivação gástrica

## Abstract

In the Roux-en-Y gastric bypass technique, classic laparoscopic surgical retractors are usually rigid, require an additional incision for its installation, or must be handled by an assistant during the surgical procedure, involving a risk of liver injury.

**Aim:**

The aim of this study was to evaluate and validate a technique of the esophagogastric junction exposure obtained by the flexible liver retractor in bariatric surgery, comparing its efficacy with the retractor classically used for this purpose.

**Methods:**

This study was performed as a randomized, open, prospective, controlled, and comparative design in patients with medical indications of bariatric surgery. The subjects were distributed in the classic (control) and flexible (test) retractor groups.

**Results:**

A total of 100 patients (n=50 control group, n=50 test group) were included. No statistically significant difference was observed in the mean duration of surgery. Regarding visibility, 100% of the patients in the flexible retractor group demonstrated an optimal visibility level, although without statistical significance concerning the classic retractor group (94%). Invariably, carrying a trocar was necessary when using the classic retractor.

**Conclusions:**

The flexible liver retractor is safe, effective, ergonomic, and inexpensive. Furthermore, it presented a satisfactory aesthetic profile, and the use of specific instruments, new adaptation curve, and training for its handling were not required.

## RESUMO - RACIONAL

Os afastadores clássicos de cirurgia laparoscópica são geralmente rígidos, necessitando de uma incisão adicional para sua instalação ou de um auxiliar para manuseio durante o ato cirúrgico e ainda, podem envolvem risco de injúria hepática.

**OBJETIVOS:**

Avaliar e validar uma técnica de exposição da junção esofagogástrica obtida pelo afastador flexível de fígado em cirurgia bariátrica comparando sua eficácia com a de afastador classicamente utilizado para este fim.

**MÉTODOS:**

Tratou-se de um estudo prospectivo, aberto, controlado e comparativo em pacientes com indicação de cirurgia, distribuídos de forma randomizada em dois grupos: clássico (controle) e afastador flexível (teste).

**RESULTADOS:**

Foram incluídos 100 pacientes (n=50 grupo controle, n=50 grupo teste), sem diferença estatística na distribuição por idade e por morbidades, havendo diferença estatística somente no gênero (grupo controle obteve proporção maior de homens, p=0,020). Em relação ao tempo médio de realização das operações, não foi constatada diferença estatística. No quesito visibilidade, verificou-se que 100% dos pacientes do grupo afastador flexível obteve nível de visibilidade ótima, porém sem significância estatística com relação ao grupo clássico (94%). Invariavelmente, foi necessário um portal a mais de trocarte quando do uso do afastador clássico.

**CONCLUSÃO:**

O afastador flexível de fígado demonstrou-se seguro, eficaz, ergonômico, de baixo custo, de perfil estético satisfatório, não requerendo instrumental específico para uso ou nova curva de adaptação e aprendizado para manuseio.

## INTRODUCTION

Obesity surgery is currently accepted as an effective treatment for morbid obesity^20^. However, bariatric surgeries are always large and complex procedures, due to the excess weight and associated diseases. Moreover, they can present postoperative morbidity (e.g., fistulas, abscesses, bleeding, and pulmonary and cardiovascular complications) that may require recovery^18^ ^,^ ^21^.

From a surgical point of view, numerous possible complications are likely to occur during the procedure, such as the gastric pouch fistula at the angle of His (esophagogastric junction). This region has lower gastric vascularization^3^ and a higher degree of difficulty in exposure during surgery, since the precision and safety of these surgeries depend on the establishment of a wide operative field.

With the advent of laparoscopy and the development of new technologies, the touch is being lost. However, the images and, therefore, visualization are better. This fact is no different from bariatric surgery, in which patients with morbid obesity can have a hypertrophic fatty left lobe of the liver, which can make it more difficult to view. Consequently, the concerning problem about the location of the angle of His under the left lobe of the liver persists. Thereby, the technique becomes even more challenging for bariatric surgeons^1^, since liver retraction is necessary to obtain a good field of vision^16^.

Most surgeons have trouble in ruling out a hypertrophic and steatotic left liver^12^. Generally, “conventional” retractors for laparoscopic surgery are rigid. Thus, an additional incision for their installation is required or they must be handled by an assistant during surgery, involving a risk of liver injury^13^. Furthermore, an additional incision increases the risk related to the wound and the number of scars.

During the 14th World Congress of the International Federation for the Surgery of Obesity (IFSO, 2009) and Metabolic Disorders in Paris, a method of hepatic retraction was presented, in which, after pneumoperitoneum installation, a straight needle was introduced into the abdominal cavity through epigastrium puncture. Then, the left lobe of the liver was initially transfixed from its parietal to the visceral surface and, using the same needle, it was again transfixed through another insertion point in the opposite direction (from visceral to parietal face). Thereafter, the needle was removed from the abdominal cavity, also by epigastric puncture from inside to outside of the abdomen. The left liver was suspended and the work area (esophagogastric junction) was adequately exposed, without the requirement for the introduction of a classic liver retractor, which would occupy an additional trocar through an additional incision. Furthermore, the need for an assistant to manipulate the instrument during the procedure was eliminated^8^. However, there was a risk of bleeding due to double liver transfixion. After observing the methods mentioned above, our team of surgeons developed a retractor (patent registration in progress) and created a new model of hepatic retraction (Moura-Babadopulos retractor) to bring the same benefits regarding the exposure of the esophagogastric junction, with added advantages such as suppression of a skin incision, no requirement for a medical assistant to manipulate the instrument, and absence of liver injury or increased risk of bleeding.

Thus, since 2009, the group of surgeons of the Núcleo do Obeso do Ceará has been regularly using the flexible liver retractor in their bariatric surgeries, presenting the model and the removal technique in Brazilian and international congresses^10^, ^11^. However, the model had not been scientifically studied for its efficacy and safety, yet.

Therefore, the objective of this study was to evaluate the efficacy and safety and to validate the flexible retractor technique in the exposure of the angle of His in the Roux-en-Y gastric bypass (RYGB) for morbid obesity.

## METHODS

All procedures involving human participants were performed under the ethical standards of the institutional and/or National Research Committee and with the 1964 Helsinki declaration and its later amendments. This study was approved by the Institutional Review Board (IRB) of the Federal University of Ceará (number: 1.482.503) and the Scientific Committee of the General Hospital Dr. César Cals. Informed consent was obtained from all participants included in this study (ClinicalTrials.gov Identifier: NCT02926885).

### Study design

This study was performed in a prospective, monocentric, open, controlled, and comparative design. A total of 100 obese patients were randomized into two different groups according to the method used for liver retraction during the surgery.

### Subjects

From April to August 2016, 100 obese patients from the Núcleo do Obeso do Ceará program (Fortaleza, Ceará, Brazil) were enrolled in the trial. The patients were of both genders and aged between 19 and 61 years, with the indication of bariatric surgery based on the criteria established by the IFSO and by the Federal Medical Council (2015).

### Operative technique

The same team performed the RYGB surgery technique by laparoscopic access to all subjects. The surgical team consisted of a surgeon responsible for performing the technique, two other surgeons working as the first and the second assistants, a surgical technologist, and two anesthesiologists.

The surgical technique performed in all investigated patients was RYGB by videolaparoscopy. After the preoperative time, a Verres needle was introduced in the hemiclavicular line-in the left hypochondrium, near the costal margin-to perform the pneumoperitoneum, which, after the constitution, had the trocars introduced. The patients were operated in a lawn chair position (approximately 30º) and a slight (approximately 10º) right lateral position for better visualization of the left hypochondrium.

A total of 4-6 trocars were used in each procedure: one 12-mm disposable trocar for stapler insertion and 4-5 permanent trocars, one being 10-mm trocar and 2-4 being 5-mm trocars (depending on the type of retractor used). The gastric pouch preparation phase began after the placement of the flexible liver retractor^®^ or the classic retractor, according to randomization. After a good visualization of the angle of His, in the gastric esophageal junction, the Fouchet tube was introduced to shape the gastric pouch (with blue cartridges). Then, the gastric remnant was isolated. A 1.5-cm diameter, 5-cm-long tubular-shaped gastric pouch was made with a volumetric capacity of approximately 60 cm³ and then a reinforcement suture was performed. After this surgical step, a 100-cm biliary limb was excluded from the duodenojejunal angle (Treitz). This segment of the jejunal, antecolic, and antegastric loop was moved toward the previously made gastric pouch and it was fixed in its inferior lateral border. Then, a manual gastrojejunal anastomosis was performed. A 120-cm alimentary loop was measured and a mechanical, white charge jejunojejunal anastomosis was made.

To prevent possible internal hernias, the space between mesocolon and mesentery (Petersen’s space) and the mesentery-mesentery space were closed.

At the end of the surgery, the test of luminous permeability and the test of impermeability of the stapling lines, sutures, and anastomosis with methylene blue were conducted. The removal of the trocars was performed under direct visualization to certify the presence or absence of bleeding through their holes.

### Flexible liver retractor

**Figure 1 -:**
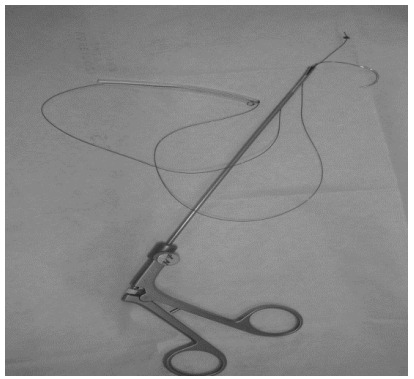
Flexible liver retractor^®^ consisting of 60-cm zero-needled silk thread glued with a 6-8-cm nelaton probe number 12.

The flexible liver retractor^®^ developed by the surgeon’s team consists of a 60-cm zero-needled silk thread with a 2.5-cm needle (Ethicon Endo-Surgery^®^), wrapped with nelaton probe number 12 and cut into 8 cm to prevent liver tissue trauma by the thread (Figure 1).

### Classic liver retractor

**Figure 2 -:**
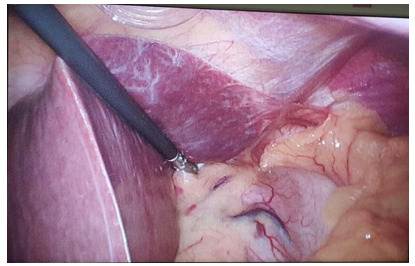
Classic liver retractor.

The classic liver retractor has been used for several years for hepatic retraction in most gastroplasty performed in Brazil. It consists of a 5-mm diameter toothed, self-static laparoscopic grasper with a rack on its cable (Figure 2).

### Study arms

The liver retraction method employed during the surgery in all patients included in this study was defined through a randomization list generated by the website http://www.randomization.com, in which each patient was allocated to one of two arms of the study, test or control arm.

#### Control arm-Classic retractor (n=50)

In the patients included in this study arm, a subxiphoid skin incision was made to introduce a 5-mm trocar. A toothed grasper was introduced through this trocar, passing between the visceral face of the left liver lobe and the stomach and fixing its teeth on the right diaphragmatic crus. Thus, the liver was kept between the grasper and the abdominal wall, providing a supermedial folding of the left liver lobe and promoting the angle of His exposure.

#### Test arm-Flexible retractor (n=50)

In this arm, the flexible liver retractor was introduced into the abdominal cavity of the patients after preparing the pneumoperitoneum and affixing the trocar. With the aid of a needle holder, the retractor needle was seized, and the right arm of the right diaphragmatic crus was transfixed near the phrenoesophageal membrane. The thread was tensioned until its casing probe was bumped into the crus. Then, the two thread ends were tensioned and the needle presented at one thread end was sectioned and removed from the abdominal cavity. The two 5-mm threads, seized by the grasper introduced through the epigastric trocar (surgeon’s left-hand-working trocar), slightly to the right side of the patient, were tensioned and removed from the abdominal cavity along with the trocar. This trocar was promptly reintroduced by the same skin incision, through which, henceforth, the threads and the trocar were passed. As the retractor was being tensioned, it shifted the left lobe of the liver anteriorly and laterally to the right, causing extensive exposure of the esophagogastric junction without traumatizing the liver tissue and without the requirement for an additional incision for the liver retractor. The system was fixed extracorporeally by a needle holder of laparotomic surgery, which seized the two threads close to the skin.

In the hepatomegaly with severe steatosis cases, with tend shaped liver, V-shaped refraction was performed. The thread was fixed by transfixion of the right crus and then in the anterior internal wall of the diaphragm, keeping the exit of both thread ends through the incision of the surgeon’s left-hand-working trocar located in the epigastrium.

At the end of the surgery, the retractor was removed from the abdominal cavity by traction on one thread end and by the surgeon’s right-hand-working trocar (12 mm).

### Assessments of variables

To evaluate the effectiveness of the flexible liver retractor, the following variables were measured and recorded during the surgical procedure:

#### Primary outcomes

**Figure 3 -:**
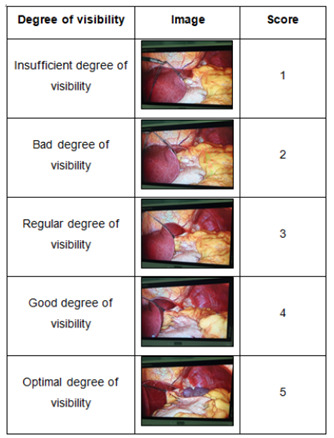
Visibility-level scale.

Visibility of the esophagogastric junction: After apposition of the classic liver retractor or the flexible liver retractor, the degree of visibility of the esophagogastric junction was evaluated and classified according to the psychometric response (at least two surgeons-first and second assistants-were always present in the surgical field) through the Likert-type scale^2^ ^,^ ^22^. The region comprising the hepatogastric ligament (pars flaccida), the small left gastric curvature, the inferior gastric antrum, the large gastric curvature (mainly the gastric bottom), the right spleen, the angle of His, and the esophagogastric junction superiorly delimited the quadrangle of interest for this study. The wide visualization of all these anatomical landmarks implied a score of 5 on the Likert-type scale (optimal degree of visibility). As the visualization of one or more structures was lost, decreasing the visualization level of the area of interest, the score was lower: 1-insufficient degree of visibility; 2-bad degree of visibility; 3-regular degree of visibility; and 4-good degree of visibility (Figure 3).

#### Secondary outcomes

These include (1) total surgery time: recorded from the beginning of the pneumoperitoneum to the last suture point on the skin; (2) time for placement of the classic liver retractor: recorded from the skin incision for the trocar insertion to the hepatic retraction and fixation of the retractor clamp teeth to the right diaphragmatic crus; (3) time for the classic liver retractor removal: recorded from the opening of the retractor clamp teeth and its removal to the skin suture, after the trocar removal; (4) time for flexible liver retractor placement: recorded from the introduction of the retractor into the abdominal cavity to the removal of both thread ends by the surgeon’s left-hand trocar and the thread external fixation with needle holder; (5) time for the flexible liver retractor removal: recorded from the opening of the needle holder that was fixing the flexible liver retractor externally until its removal from the abdominal cavity; and (6) the number of skin incisions for trocar placements.

### Statistical analysis

The quantitative variables (continuous and discrete) were initially analyzed by the Kolmogorov-Smirnov test to verify the normality of the distribution. For descriptive statistics, the mean and standard deviation (parametric data) or the median, interquartile range, and minimum and maximum values (nonparametric data) were calculated. Comparisons between the groups of patients operated with the classic retractor and the flexible retractor were performed using the t-test for unpaired variables (parametric data) or the Mann-Whitney U test (nonparametric variables). The nominal qualitative variables, expressed as absolute and relative frequency, were analyzed by Fisher’s exact test or chi-square test, as appropriate. The ordinal qualitative variables, expressed as median, interquartile range, and minimum and maximum values, were analyzed by the Mann-Whitney U test. In comparison between the two groups, the difference of means for quantitative variables or proportions for qualitative variables was determined, as well as their respective 95% confidence intervals. In all of these analyses, the significance level was set at 0.05 (5%), and a p-value <0.05 was considered statistically significant. Version 20.0 IBM SPSS Statistics for Windows^®^ (IBM Corp., Armonk, NY, USA, 2011) and version 5.00 GraphPad Prism for Windows^®^ (GraphPad Software, San Diego, California, USA, 2007) software were used to perform the statistical analysis. The GraphPad Prism for Windows^®^ software was also used for graphing.

## RESULTS

### Study population

A total of 100 obese volunteers were included in this study (n=100). In the flexible retractor group (n=50), 11 (22%) were males and 39 (78%) were females. The mean age was 36.08±10.77 years. In the classic retractor group (n=50), 23 (46%) were males and 27 (54%) were females, and the mean age was 38.10±9.77 years. The flexible retractor group showed a statistically significant difference regarding gender since a larger number of women were included in this group when compared with the classic retractor group. In other parameter comparisons, there were no significant differences between the study arms. Their demographic and clinical characteristics are shown in Table 1.

**Table 1 -:** Demographic and clinical characteristics of patients operated using the classic and the flexible retractors.

Characteristics	Arms, mean (SD)		p
	Control (classic liver retractor)	Test (flexible liver retractor)	
n	50	50	
Age (years)^a^	38.10±9.77	36.08±10.77	0.328
Gender, n (%)^b^			
Male	23 (46.00)	11 (22.00)	0.020
Female	27 (54.00)	39 (78.00)	
BMI (kg/m^2^)^a^	41.82±5.15	40.10±4.54	0.079
SAH, n (%)^b^			
Present	18 (36.00)	21 (42.00)	0.682
Absent	32 (64.00)	29 (58.00)	
Diabetes mellitus, n (%)^b^			
Present	9 (18.00)	3 (6.00)	0.121
Absent	41 (82.00)	47 (94.00)	
Osteoarthropathy, n (%)^b^			
Present	37 (74.00)	41 (82.00)	0.470
Absent	13 (26.00)	9 (18.00)	
Dyslipidemia, n (%)^b^			
Present	22 (44.00)	23 (46.00)	1.000
Absent	28 (56.00)	27 (54.00)	
Sleep apnea, n (%)^b^			
Present	25 (51.02)	19 (38.00)	0.228
Absent	24 (48.98)	31 (62.00)	
Hepatic steatosis, n (%)^c^ ^14^			
0 (absent)	8 (16.00)	12 (24.00)	0.177
1 (mild)	11 (22.00)	18 (36.00)	
2 (moderate)	19 (38.00)	13 (26.00)	
3 (severe)	12 (24.00)	7 (14.00)	
GERD, n (%)^c^ ^17^			
0 (absent )	28 (56.00)	27 (54.00)	0.201
1 (A-Los Angeles Classification)	18 (36.00)	23 (46.00)	
2 (B-Los Angeles Classification)	3 (6.00)	0 (00.00)	
3 (C-Los Angeles Classification)	1 (2.00)	0 (00.00)	

^a^t-test; ^b^Fisher’s exact test; ^c^Chi-square test; BMI, body mass index; SAH, systemic arterial hypertension; GERD, gastroesophageal reflux disease.

Bold value indicates that p-value <0.05 is statistically significant.

### Flexible liver retractor

**Figure 4 -:**
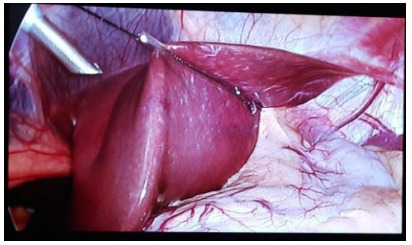
Flexible liver retractor disposed of in its most frequent form.

The flexible liver retractor disposed of in its most frequent form is observed in Figure 4. The two threads are pulled in the same direction, exiting through the same hole of the epigastrium trocar (surgeon’s left-hand-working trocar of the surgeon), displacing the left liver lobe anterolaterally, and allowing proper visualization of the angle of His.

**Figure 5:**
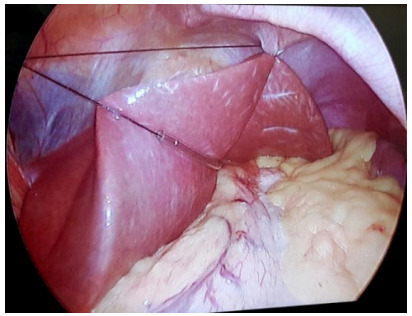
Hepatic retraction model proposed for enlarged livers.

For enlarged livers, the V-disposition hepatic refraction model was proposed to optimize the visualization of the angle of His (Figure 5).

### Surgical time

No statistically significant difference was observed between the two groups regarding the time of surgery (p=0.748, Table 2).

**Table 2 -:** Mean values and statistical comparison of surgical time, placement and removal time, and total placement and removal time of the retractor observed in the patients operated using the classic and the flexible retractors.

Time	Arms, mean (SD)		p	Difference of mean	95% CI
	Control (classic liver retractor)	Test (flexible liver retractor)			
Surgical (min)	85.70±14.06	86.66±15.66	0.748	−0.96	−6.88 to 4.96
Placement (s)	25.64±18.62	120.96±44.45	<0.001	−95.32	−108.87 to −81.77
Removal (s)	33.18±11.35	10.90±9.54	<0.001	22.28	18.11 to 26.45
Placement and removal (s)	58.82±23.53	131.86±48.45	<0.001	−73.04	−88.18 to −57.90

SD, standard deviation; 95% CI, confidence interval of 95% of the difference of mean. *Data correspond to the analysis of 50 patients in each group.

### Placement and removal time of retractor

The placement time for flexible retractor was significantly longer than for classic retractor (p<0.001). In contrast, the removal time for flexible retractor was significantly shorter than for classic retractor (p<0.001).

When the placement and removal times of both retractors were summed, the total time for the flexible retractor was significantly higher than for the classic retractor (p<0.001).

All the mean values, as well as their statistical comparisons, are observed in Table 2.

### Visibility level of the esophagogastric junction

The flexible retractor technique allowed to increase the visibility level through a second thread fixation by transfixing the right pillar and then into the anterior internal wall of the diaphragm. Thus, the visibility level in these two groups (flexible and classic retractors) was checked and observed that the visibility level provided by the flexible retractor, when fixed at only two points (in the first time of placement), was significantly lower than for classic retractor (p=0.003). When the flexible liver retractor was fixed at three points “in V” (in the second time of placement), no statistically significant difference was observed between the visibility level provided by the classic and the flexible retractors (p=0.743).

In addition, when evaluated only the first moment time of the flexible retractor placement, the proportion of patients in whom the visibility of the esophagogastric junction was graded as excellent or complete in the flexible retractor group was significantly lower (74%, p=0.012) than among the subjects from the classic retractor group (94%). However, considering the second moment, when it was necessary (13 times), all patients in the flexible retractor group obtained excellent or complete visibility (visibility level 1 or 2), without statistical significance about the classic refractor group.

### Number of skin incisions for trocar placements

In the statistical comparison of the number of skin incisions for trocar placement, the number observed in patients operated with the flexible retractor (mean of five incisions) was significantly lower than in subjects operated with the classic retractor (mean of six incisions, p<0.001).

## DISCUSSION

### Hepatic retractors

RYGB is the reference intervention for the surgical treatment of morbid obesity, and the proper exposure and visualization of the esophagogastric junction are essential for its accomplishment^5^. The effective hepatic refraction may allow easy access, adequate visualization of the operative field, and space for safe maneuvers, minimally traumatizing the tissues and anatomical planes, with the greater preservation and less manipulation of this region.

To address the above issues, many surgeons have developed techniques designed for liver retraction^4^ ^,^ ^7^ ^,^ ^8^ ^,^ ^15^ ^,^ ^16^ ^,^ ^19^. However, although there are several studies describing different types of liver retractors, there are few studies comparing the methods.

In one of these studies, Nathanson liver retractor, liver suspension tape, and V-LIST hepatic refraction methods were compared in a randomized study of 60 patients. As a result, the time required for the apposition of the V-LIST retractor was considerably longer than for the Nathanson liver retractor. However, the authors considered that their greater familiarity with Nathanson liver retractor may have interfered with this result. Invariably, for the Nathanson liver retractor, an additional skin incision was required, limiting its use for single-portal procedures. Furthermore, an increase of serum liver enzyme levels was significantly higher with Nathanson liver retractor, and more postoperative pain was observed when compared with the two other methods^6^.

In another recent study, two standard liver retractors, Nathanson and PretzelFlex, were compared using retrospective data from 167 patients (93 in the Nathanson liver retractor group and 74 in the PretzelFlex retractor group) undergoing laparoscopic RYGB. A similar duration of surgery was observed in both groups by the authors. The patients from Nathanson liver retractor group presented higher levels of alanine transaminase and C-reactive protein. The liver damage was significantly lower in the PretzelFlex retractor group (which in turn is associated with less postoperative pain and nausea) when compared with Nathanson liver retractor^9^.

### Flexible retractor × classic retractor-Relevant aspects

Upon completion of this study, all surgeries were concluded with an adequate esophagogastric junction visibility level, suggesting noninferiority of the flexible liver retractor when compared with the classic liver retractor. Nevertheless, the following relevant aspects should be considered: the flexible retractor, by requiring more maneuvers (including needle manipulation), demanded more time for placement, leading to a statistically significant difference when compared with the classic retractor, which has simpler handling during its placement; for removal, however, there was a difference in favor of the flexible retractor. While it only needs to be pulled out of the abdominal cavity after opening the needle holder that supports it on the outside, removal of the classic retractor is only completed after incised skin suture. Finally, the total surgical time was similar for both retractors.

### Positive points of the flexible retractor

Despite the time difference, when comparing total operative time, a surgical parameter of real interest, no statistical relevance in favor of either retractor was observed, which may indirectly indicate the adequate visualization offered by the flexible retractor. Invariably, carrying an extra trocar when using the classic retractor was always necessary, as it requires a skin hole for its placement.

In case of minimally invasive surgeries, when the same or the best result is sough, but causing the least possible damage related to the inflammatory response to trauma, or related to the possibility of less bleeding, incisional hernias, infection, or hypertrophic healing than each new incision may represent, this is an undoubted advantage of using the flexible liver retractor.

Another point to be considered, although it has not been shown, is a liver injury, which can be minimized by the use of a flexible retractor, as it shapes to the liver surface, having less likely to fracture it than the rigid retractors, like those used in this study as a control. Also important is the spatial characteristic of the flexible retractor, which, being totally intracavitary, is not susceptible to collisions with the grasper or the arms of surgeons or assistants. This characteristic could become even more important if surgeries performed with robotic assistance are considered, in which the robot’s arms may collide with the patient or with a retractor rod that protrudes out of the patient, leading to accidents. Trocars incisions may be foci of infection, bleeding, pain, hypertrophic scars, or wall hernias that can complicate bowel obstruction, which, although rare, are described even with 5-mm punctures. Thus, during laparoscopic procedures, trocars are used through which the instruments are passed and, as far as possible, the smallest number of them are used as long as the safety of the surgical gesture is not compromised. Although, even with the requirement for one less incision in the patients of the flexible retractor group, a postoperative routine change was not observed when compared with the classic retractor group. Once placed, the classic retractor leaves little repositioning alternative to optimize the esophagogastric junction visibility level. If it is inadequate enough to interfere with the safety of the procedure, it can only be corrected with the introduction of a new retractor clamp by an additional trocar size. Also, as in clinical practice, occupying one of the auxiliary surgeon’s hands during the main time of surgery to maintain adequate liver refraction will prevent the use of an auxiliary trocar size, which would further minimize the number of incisions in the wall. However, the auxiliary would work with only one hand.

By avoiding one more puncture, the auxiliary help capacity is limited. In contrast, the flexible retractor allows variable positioning of one of its handles (one of its constituent threads), which provides versatility for use on different shapes of livers.

The flexible retractor presents the technical conditions to be validated as a liver retraction instrument for adequate and safe exposure of the esophagogastric junction in bariatric surgeries and in other upper abdomen procedures in which the liver makes visibility difficult. In addition, because this retractor allows exposure of the upper abdomen without the need for incisions for this purpose, it meets the characteristics of minimally invasive surgery equipment and can be used in single-portal operations. Also, because it does not require external fixation mechanisms, it may be an alternative to retractors currently used in robotic surgery because of the minimal risk of collision with the robot arms.

This study has some limitations, especially regarding the information recording of the surgical process, such as the amount of drugs required for analgesia, registration of complications during and after surgery, evaluation of pain in recovery, and length of hospital stay. Another control group has also been used employing other liver retractors, such as Nathanson liver retractor.

## CONCLUSION

The flexible liver retractor was safe, effective, ergonomic, and inexpensive, with a satisfactory aesthetic profile. Furthermore, the use of specific instruments or adaptation curve and training were not required, making it suitable to be used as an alternative to retractors currently available.

## References

[1] Ahmad A, Arellano JJ, Agarwala A, Ahmad Z, Ahmad Z (2016). A percutaneous technique of liver retraction in laparoscopic
bariatric & upper abdominal surgery. Surg Obes Relat Dis.

[2] Allen I., Seaman C. (2007). Statistics Roundtable: Likert Scales And Data Analyses.

[3] Batista Marchesini J (2007). A Safer and Simpler Technique for the Duodenal Switch: To the
Editor. Obes Surg.

[4] Galvani CA, Choh M, Gorodner MV (2010). Single-incision sleeve gastrectomy using a novel technique for
liver retraction. JSLS.

[5] Garrido AB, Elias AA, Oliveira MR, Ito RM, Shirozaki HY (2015). Tratado de obesidade.

[6] Goel R, Shabbir A, Tai CM, Eng A, Lin HY, Lee SL, Huang CK (2013). Randomized controlled trial comparing three methods of liver
retraction in laparoscopic Roux-en-Y gastric bypass. Surg Endosc.

[7] Hamzaoglu I, Karahasanoglu T, Aytac E, Karatas A, Baca B (2010). Transumbilical totally laparoscopic single-port Nissen
fundoplication: a new method of liver retraction: the Istanbul
technique. J Gastrointest Surg.

[8] Huang CK, Houng JY, Chiang CJ, Chen YS, Lee PH (2009). Single incision transumbilical laparoscopic Roux-en-Y gastric
bypass: a first case report. Obes Surg.

[9] Midya S, Ramus J, Hakim A, Jones G, Sampson M (2020). Comparison of Two Types of Liver Retractors in Laparoscopic
Roux-en-Y Gastric Bypass for Morbid Obesity. Obes Surg.

[10] Moura LG, Feitosa HC, Machado FHF, Babadopulos RF, Feijó FC, Fernandes SD (2012). Minimizing portals with liver malleable retractor in bariatric
laparoscopic acess, N.O.T.E.S. robotic. P.21 - Poster Sessions.

[11] Moura-Júnior LG, Castro-Filho HF, Machado FH, Babadopulos RF, Feijó FD, Fernandes SD (2014). Ports minimization with mini-port and liver flexible retractor:
an ergonomic and aesthetic alternative for single port in laparoscopic
gastric bypass. ABCD. Arq Bras Cir Dig.

[12] Nguyen NT, Longoria M, Gelfand DV, Sabio A, Wilson SE (2005). Staged laparoscopic Roux-en-Y: a novel two-stage bariatric
operation as an alternative in the super-obese with massively enlarged
liver. Obes Surg.

[13] Pasenau J, Mamazza J, Schlachta CM, Seshadri PA, Poulin EC (2000). Liver hematoma after laparoscopic nissen fundoplication: a case
report and review of retraction injuries. Surg Laparosc Endosc Percutan Tech.

[14] Saadeh S, Younossi ZM, Remer EM, Gramlich T, Ong JP, Hurley M, Mullen KD, Cooper JN, Sheridan MJ (2002). The utility of radiological imaging in nonalcoholic fatty liver
disease. Gastroenterology.

[15] Saber AA, El-Ghazaly TH (2009). Early experience with single incision transumbilical laparoscopic
adjustable gastric banding using the SILS Port. Int J Surg.

[16] Sakaguchi Y, Ikeda O, Toh Y, Aoki Y, Harimoto N, Taomoto J, Masuda T, Ohga T, Adachi E, Okamura T (2008). New technique for the retraction of the liver in laparoscopic
gastrectomy. Surg Endosc.

[17] Sami SS, Ragunath K (2013). The Los Angeles classification of gastroesophageal reflux
disease. Video journal and Encyclopedia of GI Endoscopy.

[18] Santoro S, Aquino CGG, Mota FC, Artoni RF (2020). Does evolutionary biology help the understanding of metabolic
surgery? A focused review. Arq Bras Cir Dig.

[19] Shibao K, Higure A, Yamaguchi K (2011). Disk suspension method: a novel and safe technique for the
retraction of the liver during laparoscopic surgery (with
video). Surg Endosc.

[20] Stein J, Stier C, Raab H, Weiner R (2014). Review article: The nutritional and pharmacological consequences
of obesity surgery. Aliment Pharmacol Ther.

[21] Stoll A, Rosin L, Dias MF, Marquiotti B, Gugelmin G, Stoll GF (2016). Early Postoperative complications in Roux-en-Y gastric
bypass. Arq Bras Cir Dig.

[22] SurveyMonkey (2017). Likert Scale: What It Is & How To Use It | Surveymonkey.

